# Influence of the Impregnation Technique on the Release of Esomeprazole from Various Bioaerogels

**DOI:** 10.3390/polym13111882

**Published:** 2021-06-06

**Authors:** Milica Pantić, Katja Andrina Kravanja, Željko Knez, Zoran Novak

**Affiliations:** 1 Faculty of Chemistry and Chemical Engineering, Laboratory of Separation Processes and Product Design, University of Maribor, Smetanova 17, 2000 Maribor, Slovenia; milica.pantic1@um.si (M.P.); katja.kravanja1@um.si (K.A.K.); zeljko.knez@um.si (Ž.K.); 2 Faculty of Medicine, University of Maribor, Taborska Ulica 8, 2000 Maribor, Slovenia

**Keywords:** bioaerogels, polysaccharides, esomeprazole, controlled release, supercritical impregnation

## Abstract

The presented study shows the possibility of using bioaerogels, namely neat alginate, pectin, chitosan aerogels, and alginate and pectin aerogels coated with chitosan, as drug delivery systems for esomeprazole. Two different techniques were used for the impregnation of esomeprazole: Supercritical impregnation, and diffusion via ethanol during the sol-gel synthesis. The prepared samples were characterized by employing N_2_ adsorption-desorption analysis, TGA/DSC, and FTIR. The achieved loadings were satisfactory for all the tested samples and showed to be dependent on the technique used for impregnation. In all cases, higher loadings were achieved when impregnation via diffusion from ethanol was used. Extensive release studies were performed for all impregnated samples. The in vitro dissolution profiles were found to be dependent on the carrier and impregnation method used. Most importantly, in all cases more controlled and delayed release was achieved with the bioaerogels compared to using pure esomeprazole.

## 1. Introduction

Over the years, the pharmaceutical industry has made great efforts to investigate the physiochemical properties of drugs. An important factor, however, is the bioavailability of drugs due to physiological and pathological processes in the human body that influences the release and absorption of the active ingredient from its carrier [1]. Therefore, it is important to investigate controlled drug release systems in order to incorporate the right dosage of the drug into the body, increase its efficiency, prevent drug degradation, and its negative side effects on the body [2]. Various studies have shown the effectiveness of achieving controlled drug release by particle size reduction, modification of crystalline structure, and the use of emulsion, dispersion or lipid delivery systems, among other approaches [1,3,4]. One method that shows great potential for increasing the bioavailability of the drugs is their incorporation into aerogels [5,6].

Polysaccharide aerogels, such as pectin, alginate, and chitosan, are particularly attractive for use as oral drug carriers due to their biocompatibility, biodegradability, affordability, and non-toxicity [7]. Pectin is mainly found in fruits as a structural component of their primary cell walls [8]. It consists of galacturonic acid monomers, which are connected to a linear structure by a α-(1,4)-glycosidic bond [9]. Alginates are a family of copolymers consisting of α-L-guluronic acid (G blocks) in β-D-manuronic acid (M blocks) monomers linked via 1,4-glycosidic bonds. The source of most alginates is brown algae (Phaeophyceae) [10,11]. Both pectin and alginate have excellent gelling properties and are readily soluble in water. Moreover, pectin and alginate aerogels are promising candidates for impregnation of poorly water-soluble drugs in order to improve their bioavailability [10]. Furthermore, recent studies have shown that in order to prevent the rapid release of some drugs a more controlled release could be achieved by coating the gels [12,13,14,15]. Chitosan consists of 2-acetamide-2-deoxy-beta-D-glucopiranose and 2-amino-2-deoxy-beta-D-glucopirnose monomers linked together by 1,4-glycosidic bonds. It is obtained by deacetylation of chitin, a compound found in insects, crustaceans, and fungi. It is a polycation consisting of protonated amino groups and is therefore only soluble in dilute acids with a pH below 6. The latter can be used as an advantage for slowing down and achieving more controlled release of impregnated drugs when used as a coating for water-soluble gels, such as pectin and alginate [12,16,17].

Esomeprazole is a drug that reduces the production of stomach acid by inhibiting a proton pump. Therefore, it is used to treat gastroesophageal reflux disease, Zollinger-Ellison syndrome, peptic ulcers, and other symptoms or diseases caused by excessive excretion of stomach acid [18,19]. It is generally used and sold under the name Naxium, produced by AstraZeneca. There have also been attempts at using strontium (Sr) capsules for achieving the delayed release of esomeprazole. However, esomeprazole has limited absorption by the body [20,21,22]. There is currently a very limited number of studies in the literature concerning esomeprazole and esomeprazole impregnated into carriers. Some of these carriers include locust bean and xanthan gum polymers applied in a spray drying process [23], pH-sensitive starch hydrogels [24], and zinc (Zn) solid dispersion for delayed release [25]. The target for the release of the esomeprazole is the stomach. However, some of the focal syndromes and diseases that this drug treats involve also the intestines. The idea of this study was thus to create a controlled delivery system that will be able to prolong and partially deliver the drug to the upper part of the small intestine, as well. The objective was to improve the bioavailability of esomeprazole by impregnating it in pectin, alginate, and chitosan aerogels using two different methods: Supercritical impregnation with carbon dioxide, and diffusion from ethanol during sol-gel synthesis. In addition, pectin and alginate aerogels were coated with chitosan in the hope of accomplishing a more controlled release of the drug. The release behavior of the esomeprazole was extensively investigated to see the influence of impregnation technique and carrier on the release profile.

The presented study focuses on reducing the release and consequently dissolution, with the focal drug in this case being esomeprazole. The results of the experiments show that this treatment can significantly improve the bioavailability of esomeprazole.

## 2. Materials and Methods

### 2.1. Materials

**Figure 1:**
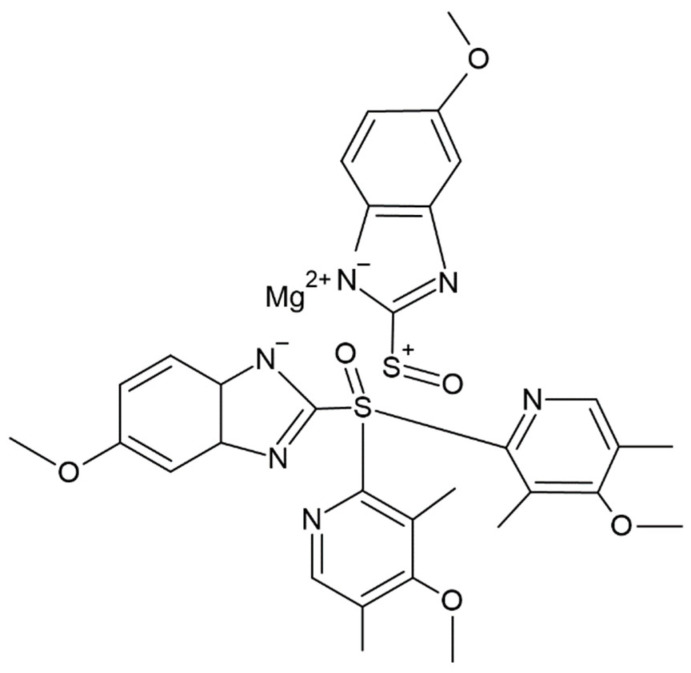
Structural formula of esomeprazole magnesium trihydrate.

Three different polysaccharides were used for preparation of the gels, and then the bioaerogels. Alginic acid sodium salt from algae (CAS 9005-38-3) and chitosan with medium molecular weight (CAS 9012-76-4) were purchased from Sigma-Aldrich (St. Louis, MO, USA), while pectin from citrus (CAS 9000-69-5) was purchased from Tokyo Chemical Industry, TCI Europe (Zwijndrecht, Belgium). For the dissolution of polysaccharides, distilled water was used in the case of alginate and pectin, while chitosan was dissolved in acetic acid, CH_3_COOH (Fisher Scientific, Pittsburg, PA, USA; purity ≥ 98%). Sodium hydroxide, NaOH (Merck, Darmstadt, Germany; purity ≥ 98%) was used for preparation of coated composite aerogels. Absolute ethanol, C_2_H_5_OH (Merck, Darmstadt, Germany) was employed for the gelation of polysaccharide solutions, while carbon dioxide, CO_2_ (Messer, Ruše, Slovenia; purity 99.5%) was used for supercritical drying of obtained gels. Esomeprazole magnesium trihydrate, C_34_H_42_MgN_6_O_9_S_2_ (Xi’an Health Biochemical Technology Co., Xi’an, Shaanxi, China; purity 99.0%) was used as a model drug for the impregnation experiments with bioaerogels and the subsequent in vitro dissolution tests. Its structural formula is shown in Figure 1.

Hydrochloric acid, HCl (Merck, Darmstadt, Germany; 37%); potassium phosphate monobasic, KH_2_PO_4_ (Merck, Darmstadt, Germany; purity ≥ 98%), and sodium hydroxide, NaOH (Merck, Darmstadt, Germany; purity ≥ 98%), were employed for the preparation of simulated gastric (SGF) and simulated intestinal fluid (SIF) for the in vitro dissolution tests. SGF (HCl) with pH = 1.2 was prepared by diluting 8.3 mL of 37% HCl to 1000 mL with Milli-Q water. The phosphate buffer solution (SIF) with pH = 6.8 was prepared by mixing 250 mL of 0.2 M KH_2_PO_4_ and 112 mL of 0.2 M NaOH and diluted to 1000 mL with Milli-Q water.

### 2.2. Methods

#### 2.2.1. Preparation of Alginate, Pectin, and Chitosan Gels

Neat samples of alginate, pectin, and chitosan were prepared by ethanol induced gelation [26]. In this, 2% (*w*/*w*) alginate and pectin solutions were prepared by dissolving a weighted amount in distilled water. In the case of chitosan, 1.5% solution (*w*/*w*) was prepared in 0.2 M CH_3_COOH. In the next step, the solutions were transferred into petri dishes, soaked into ethanol, and left overnight for gelation. Once the gels were formed they were cut precisely into regular tablets.

#### 2.2.2. Preparation of Alginate and Pectin Gels Coated with Chitosan

Additionally, alginate and pectin gels were prepared as composites with chitosan. The coating procedure [12] consisted of adding a chitosan coating over a core, made of alginate or pectin. Pectin and alginate cores were prepared in the same manner as neat gels, following the procedure described above. To add a coating, either alginate or pectin cores were poured into the 1.5% chitosan solution (*w*/*w*). Soaked cores covered with chitosan solution were promptly transferred to 2 M NaOH in ethanol to attach the coating over the core. NaOH was used as a crosslinker since it triggers the gelation of chitosan. In order to remove the excess NaOH, the samples were then washed with ethanol.

#### 2.2.3. Supercritical Drying of Gels

A supercritical drying technique was employed to obtain aerogels prepared from neat and composite coated gels. Therefore, all the samples were subjected to already optimized conditions for polysaccharide gel drying, at 120 bar, 40 °C for 6 h.

#### 2.2.4. Impregnation of Esomeprazole

To impregnate esomeprazole into the aerogels, two different methods were applied considering the solubility of esomeprazole in various solvents. Esomeprazole is slightly soluble in water (0.353 mg/mL) and belongs to BCS Class II [27,28].

Firstly, diffusion via ethanol (DIF) was performed simultaneously with sol-gel synthesis [29], as esomeprazole is soluble in ethanol (1 mg/mL) [30]. Once the molecules of esomeprazole are dissolved, they diffuse inside the gel pore network via ethanol from a saturated solution. The other technique involves the post-treatment of already synthesized aerogels (SCI) [31]. For this purpose, a high-pressure impregnation cell was used. Bioaerogels were placed at the bottom of the impregnation cell, while the esomeprazole was placed above. Both bioaerogels and esomeprazole were placed into filter bags, to ensure that only the dissolved esomeprazole diffused inside the porous network. CO_2_ was introduced into the high impregnation cell, and first came across the esomeprazole, dissolving it and bringing it into the bioaerogels. The experiment was performed in batch mode for 24 h at 35 ± 1 °C and 110 ± 10 bar. The solubility of esomeprazole in CO_2_ varies from 0.034 to 5.599 mg/mL, depending on the conditions used (*p* = 120–270 bar, T = 35–65 °C) [32]. Finally, the system was slowly depressurized (3 bar/min) to avoid possible damage to the bioaerogels’ porous networks.

The final loadings were determined by extraction of impregnated esomeprazole from the bioaerogels. The loaded bioaerogels were placed in a glass beaker containing SGF for a few hours (either until complete dissolution in the case of alginate and pectin or until equilibrium was achieved in the case of chitosan). The concentration of esomeprazole was calculated using calibration curves after determining the absorbance in the solutions using a Cary 50 Probe UV spectrophotometer operated at 301 nm. The determined loadings were also verified using ethanol as the dissolving medium.

#### 2.2.5. In Vitro Dissolution Tests

In vitro dissolution tests were performed for pure esomeprazole and all bioaerogels impregnated with esomeprazole using either diffusion via ethanol or supercritical impregnation. All the tests were performed in combined media: 2 h in SGF at pH = 1.2 and 22 h in SIF at pH = 6.8. The samples were first placed in SGF for 2 h and immediately transferred to SIF for the next 22 h.

The experiments were carried out on the Farmatester 3 (Dema, Ilirska Bistrica, Slovenia), USP II apparatus, following USP standards [33] in triplicate. The conditions used for performing the test were 37 ± 0.5 °C, 900 mL of dissolution media with 50 rpm speed of rotation. At predetermined time periods (for SGF: 5, 15, 30, 60, 90, and 120 min and for SIF: 5, 15, 30, 60, 90, 120, 180, 240, 300, 360, 420, and 1440 min) aliquots of 2 mL for each sample were withdrawn and afterwards an equal volume of fresh dissolution medium was added back to maintain a constant volume.

To determine the absorbance of esomeprazole, the samples were subjected to the assay using a Cary 50 Probe UV spectrophotometer (Agilent Technologies, Santa Clara, CA, USA) operated at 301 nm. The concentrations were calculated using calibration curves made in SGF and SIF in the same way as explained above.

#### 2.2.6. Characterization Methods

The textural properties of prepared aerogels were tested by employing the adsorption-desorption analysis. ASAP 2020MP instrument (Micromeritics, Norcross, GA, USA) was used to determine the specific surface areas of prepared bioaerogels. The samples were first exposed to a degassing procedure under a vacuum until a stable pressure is obtained. The samples were then analyzed using N_2_ as the adsorptive gas. For recalculating the specific surface area, the Brunauer-Emmett-Teller (BET) method was used. The measured adsorption isotherms were used for presentation of the bioaerogels’ adsorption capacities.

Impregnated bioaerogels and esomeprazole were characterized by Fourier transform infrared spectroscopy on IRAffinity-1s apparatus (Shimadzu, Kyoto, Japan) through applying the ATR-IR method for the bioaerogel samples and KBr method for esomeprazole. The measured spectra of neat alginate, pectin and chitosan aerogels as well as alginate and pectin aerogels coated with chitosan were used to identify characteristic adsorption bands of the polysaccharides and impregnated esomeprazole.

The thermal stability of esomeprazole and impregnated bioaerogel samples was determined by thermogravimetry and differential scanning calorimetry analysis, carried out on a TGA/DSC1 apparatus (Mettler Toledo, Columbus, OH, USA). The TGA and DSC analyses were performed simultaneously, using a 30–600 °C temperature range, with a 10 °C per minute heating rate under an air atmosphere.

## 3. Results

### 3.1. Characterization of Materials

#### 3.1.1. N_2_ Adsorption-Desorption Analysis 

The N_2_ adsorption-desorption analysis was applied to determine the specific surface areas of all the samples used in the impregnation process. As presented in Table 1, the highest specific surface area was achieved in the case of pectin aerogels, reaching 501 m^2^/g. Chitosan aerogels followed this, with a specific surface area of 431 m^2^/g, while the alginate aerogels had the lowest, at 220 m^2^/g. When pectin and alginate aerogels were coated with chitosan, their specific surface areas both reduced, reaching 314 and 248 m^2^/g, respectively. The porous structure was slightly affected by the preparation process, causing smaller specific surface areas. It is important to note that the coated aerogels maintained their porous structures and their adsorption capacities.

The reported specific surface areas differ depending on the polysaccharide, composition, and preparation technique used. Specific surface areas for alginate are usually in the range between 130–550 m^2^/g [4,7,34], while for pectin they are between 250–630 m^2^/g [35,36,37] and for chitosan 110–840 m^2^/g [17,38]. All the tested samples from this study are within this range. In the case of pectin and chitosan, the specific surface areas are closer to the upper limits, showing that they are potentially very effective drug carriers. 

**Figure 2:**
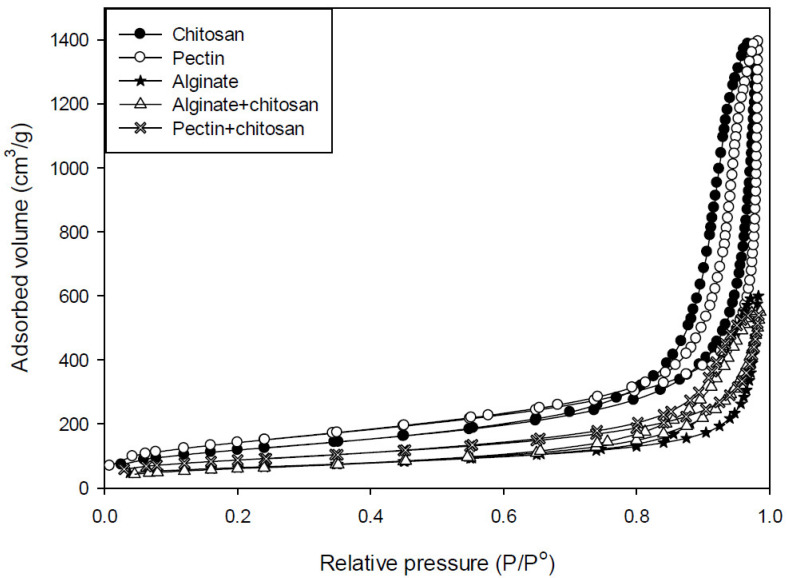
Adsorption isotherms of pectin, alginate, chitosan, and their composites.

The N_2_ adsorption-desorption analysis was further employed to investigate the adsorption capacities of the used bioaerogels. This property is crucial when considering impregnation of the samples with active ingredients. With high adsorption capacities, aerogels can take a significant amount of active ingredients into their interiors. As shown in Figure 2, pectin and chitosan aerogels have quite high adsorption capacities, showing very good abilities for impregnation of active ingredients. In contrast, alginate and coated composite bioaerogels have lower adsorption capacities. These results are in good agreement with the specific surface areas. The higher the adsorption capacities, the higher the specific surface area, and vice versa. Lastly, the shapes (type IV) of the adsorption isotherms classify the materials as mesoporous.

#### 3.1.2. Thermal Analysis (Thermogravimetry and Differential Scanning Calorimetry Analysis)

Thermogravimetry (TGA) and differential scanning calorimetry (DSC) analyses were performed simultaneously. The objective was to determine the thermal properties of pure esomeprazole and bioaerogels impregnated with esomeprazole. The obtained results are given as TGA and DSC curves, presented below.

**Figure 3:**
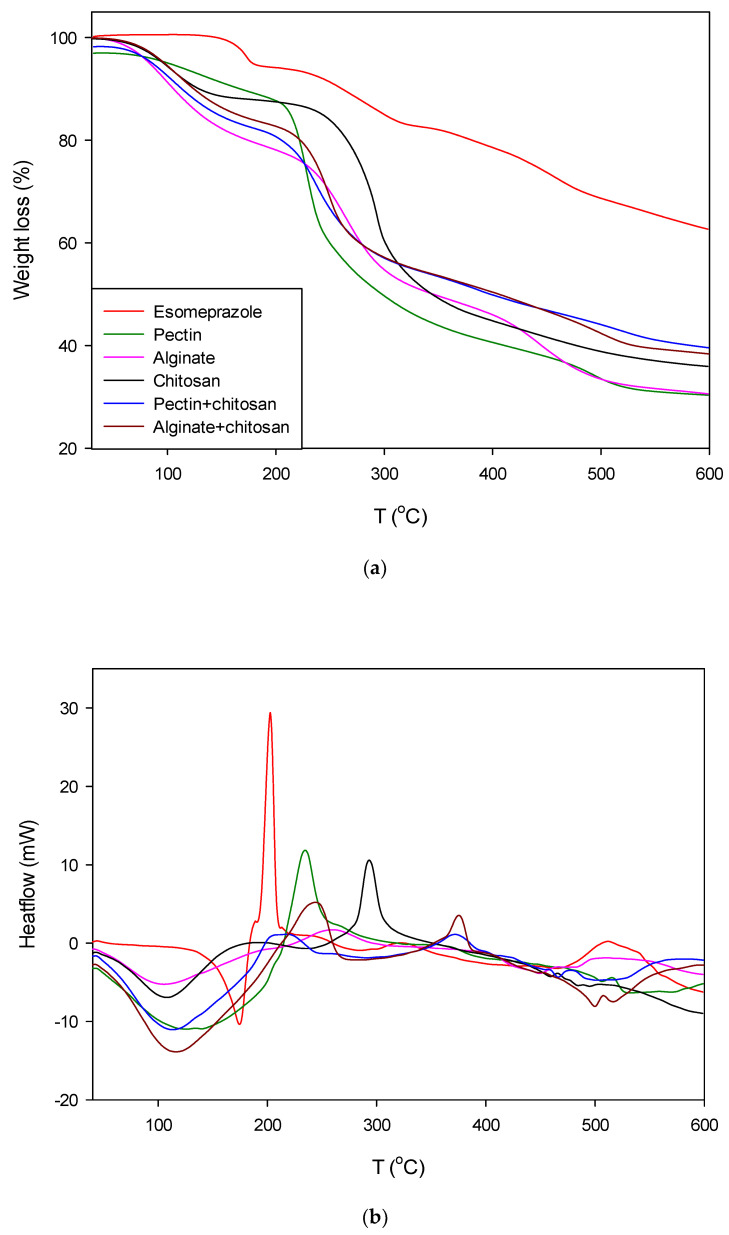
(**a**) TGA curves for esomeprazole and pectin, alginate, chitosan, and their composites impregnated with esomeprazole, (**b**) DSC curves for esomeprazole and pectin, alginate, chitosan, and their composites impregnated with esomeprazole.

TGA curves for the tested samples are shown in Figure 3a. The tested temperature range was from room temperature up to 600 °C. It can be seen that esomeprazole shows the slowest degradation for all the tested range, having degradation of 37%. Pectin and alginate aerogels impregnated with esomeprazole show a similar behavior, with 70% degradation. Chitosan aerogels impregnated with esomeprazole, on the other hand, showed 64% degradation. As such, it can be seen that the chitosan part improved the stability of the composite bioaerogels. Therefore, alginate and pectin aerogels coated with chitosan have lower degradation compared to the neat alginate and pectin aerogels, with the former pair showing degradation of 60%.

Figure 3b presents the DSC curves for esomeprazole, pectin, alginate, and chitosan aerogels impregnated with esomeprazole, along with those for the pectin and alginate aerogels coated with chitosan impregnated with esomeprazole. For the DSC curves, only the temperature range up to 450 °C is presented, since for higher temperatures no additional peaks were visible. Pure esomeprazole shows two peaks, and this pattern indicates exothermic melting by decomposition. Firstly, the endothermic peak at 175 °C represents its melting point, while the exothermic peak at 202 °C represents its decomposition temperature [39]. In the case of pectin, alginate, and chitosan aerogels impregnated with esomeprazole, the DSC thermograms exhibit an endothermic peak and an exothermic peak. Endothermic peaks are correlated to the elimination of loosely bound water in the samples. The exothermic peak is visible for each of these, representing the decomposition of each polysaccharide. The temperature for each polysaccharide differs, depending on the decomposition point. In the case of pectin this peak occurs at 237 °C, in the case of alginate at 260 °C, while in the case of chitosan at 293 °C, indicating the degradation of each polysaccharide. Compared to neat bioaerogels, alginate and pectin aerogels coated with chitosan have one additional exothermic peak. In the case of alginate, these peaks occur at 248 and 372 °C, and for pectin at 218 and 372 °C. These two peaks come from the composite structure of these aerogels, possessing two different polysaccharides. Lastly, the characteristic peaks of esomeprazole are not visible in the DSC thermograms of aerogels due to the low quantity of esomeprazole in the analyzed samples. Another possibility is the change of esomeprazole from crystalline to amorphous state when impregnated into aerogels, where the position of the peaks could also be shifted and overlapped with the ones from the polysaccharides [40].

#### 3.1.3. Fourier Transform Infrared Spectroscopy

**Figure 4:**
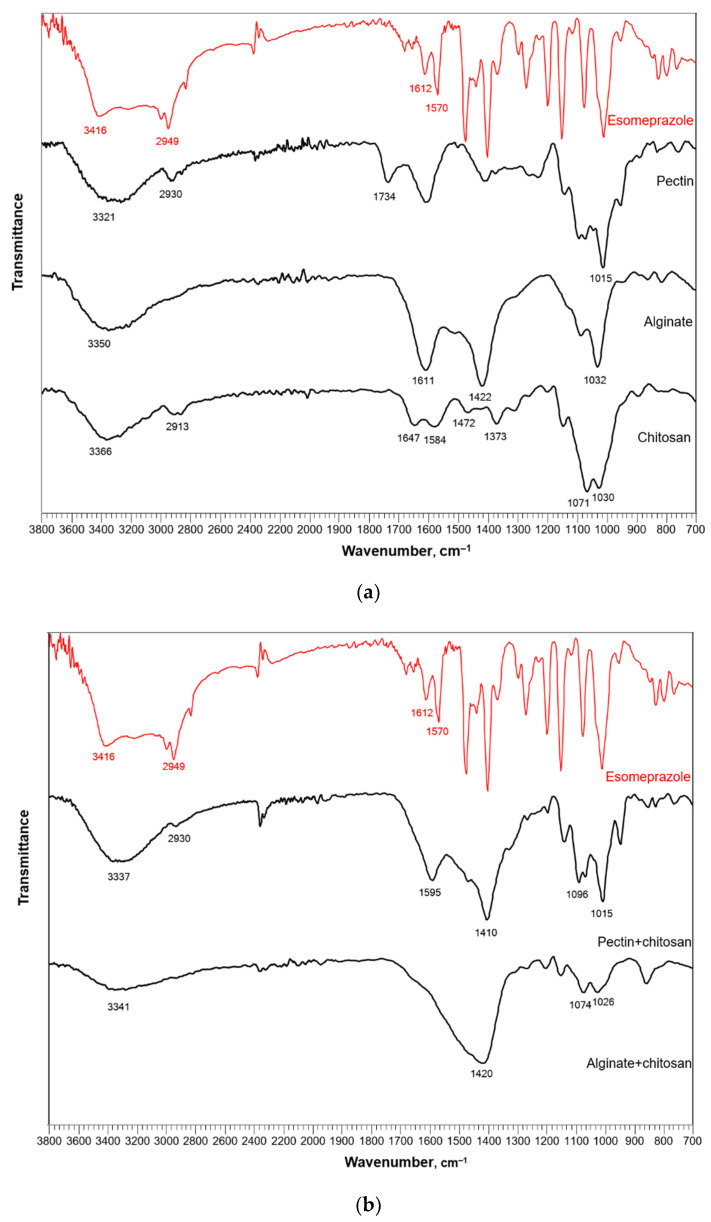
(**a**) FTIR spectra of pure esomeprazole and pectin, alginate, and chitosan aerogels impregnated with esomeprazole, (**b**) FTIR spectra of pure esomeprazole and pectin and alginate aerogels coated with chitosan impregnated with esomeprazole.

Fourier transform infrared spectroscopy (FTIR) was performed to determine the spectra and characterization of pure esomeprazale and bioaerogels impregnated with esomeprazole. Figure 4a shows the spectra of pure esomeprazole and pectin, alginate, and chitosan aerogels, while Figure 4b shows the spectra of esomeprazole and alginate and pectin aerogels coated with chitosan. The spectrum of pure esomeprazole is used in both figures, for comparison with all impregnated samples.

The measured IR spectrum of esomeprazole corresponds to the one in the literature [27]. The broad peak corresponds to the C=N group. The peak at 3417 cm^−1^ is characteristic of the N–H group, while the one at 2949 cm^−1^ of the C=C group. Peaks at 1612 and 1570 cm^−1^ indicate the presence of carbonyl groups.

The characteristic peaks for both pectin and alginate are visible in the spectra of pectin and alginate aerogels impregnated with esomeprazole. First, the broad peak at 3321 cm^−1^ for pectin and 3350 cm^−1^ for alginate represent the hydroxyl O–H groups. Furthermore, the bands for alginate at 1611 and 1422 cm^−1^ show asymmetric and symmetric stretching of the carboxyl COO^–^ groups. On the other hand, the peak at 1734 cm^−1^ for the pectin aerogel shows the esterified carboxyl groups. Finally, the peaks at 1015 cm^−1^ for pectin and 1032 cm^−1^ for alginate appear due to the C–O groups with the saccharide structure. The spectrum for the chitosan aerogels also corresponds to that in the literature. A strong band at 3366 cm^−1^ presents the stretching of N–H and O–H and the intramolecular hydrogen bonds. The band at 2913 cm^−1^ can be attributed to C–H symmetric stretching. Peaks at 1647 and 1584 cm^−1^ are characteristic for N-H and C–N amid the functional groups, while the peaks at 1472 and 1373 cm^−1^ represent CH_2_ bending and CH_3_ symmetrical deformations. Finally, the peaks at 1071 and 1030 cm^−1^ are characteristic of C–O stretching.

When comparing the spectra for bioaerogels impregnated with esomeprazole with that for pure esomeprazole, for both pectin and chitosan a characteristic peak which may be attributed to the C=N group appears at 2930 cm^−1^ for pectin and 2913 cm^−1^ (overlapping with the C–H symmetric stretching from chitosan). The alginate spectrum does not have a clear peak in this position. However, the mild curvature at this position is visible and could be attributed to the presence of esomeprazole. Other characteristic peaks for esomeprazole, at 1612 and 1570 cm^−1^, are on the other side, overlapping with the peaks of bioaerogels.

The spectra of pectin and alginate coated with chitosan are slightly changed compared to the pectin and alginate aerogels without chitosan coating, as shown in Figure 4b. In this case, some peaks are shifted and some of them are overlapping, proving the presence of both pectin or alginate and chitosan. The presence of esomeprazole is indicated by the peak in the area close to 2930 cm^−1^, faintly visible for the alginate aerogel coated with chitosan (the same as for the neat alginate aerogels). Other characteristic peaks of esomeprazole are most likely overlapping with the peaks of pectin, alginate, and chitosan.

Lastly, all impregnated aerogels keep the characteristic peaks for the neat polysaccharides used for their production. This is important to emphasize, as it leads to the conclusion that the carriers, in this case bioaerogels, do not chemically change through the process of impregnation (no matter the technique used). The impregnated drug esomeprazole enters the interior of the aerogels, leaving the chemical structure of the polysaccharide unchanged by physically bonding to its network.

### 3.2. Esomeprazole Loadings

Table 2 presents the achieved loadings for pectin, alginate, and chitosan aerogels and pectin and alginate aerogels coated with chitosan. As expected, the highest loadings regardless of the loading procedure were achieved in the case of pectin and chitosan, at 19.5 and 22%, respectively. The lowest, on the other hand, was obtained for the coated pectin and alginate aerogels, at 2.5 and 8.5%, respectively. These results are in good agreement with the obtained specific surface areas and adsorption capacities. The statement that the higher adsorption capacities, the greater ability for the loading with active ingredients is thus confirmed.

Furthermore, the level of the loadings was defined by the loading procedures. Generally, higher loadings were achieved when the diffusion method was used for the impregnation of esomeprazole. This effect was observed in all cases. The differences vary between 0.5 to 4.5%. The greatest difference was found for chitosan aerogels.

The solubility of esomeprazole in ethanol is slightly higher than that in supercritical carbon dioxide, at 1 and 0.73 mg/mL under the impregnation conditions, respectively [30,32]. This could be the reason for the higher loadings that were achieved when using diffusion from ethanol as the impregnation procedure.

**Figure 5:**
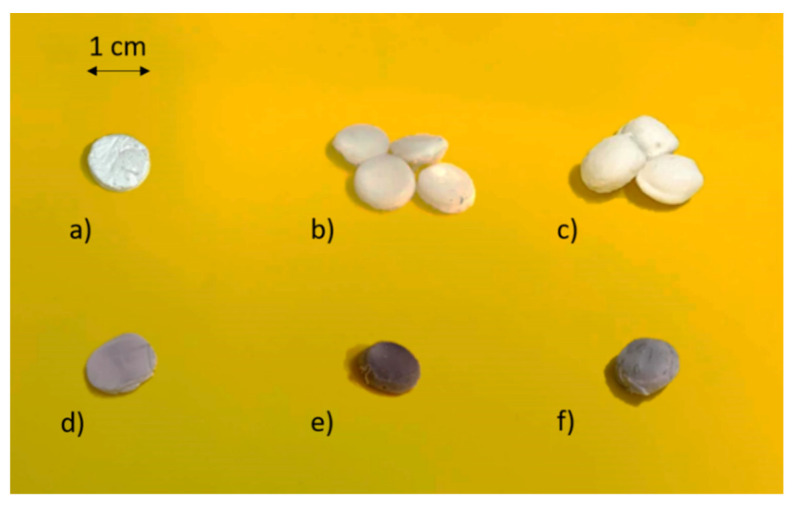
Non-impregnated and impregnated aerogels: (**a**) Alginate aerogels, (**b**) chitosan aerogels, (**c**) alginate aerogels coated with chitosan, (**d**) impregnated alginate aerogels, (**e**) impregnated chitosan aerogels, and (**f**) impregnated alginate aerogels coated with chitosan.

Figure 5a–f shows blank, non-impregnated bioaerogels and the same bioaerogels when impregnated with esomeprazole. By impregnating esomeprazole, the color of the neat bioaerogels changes to purple, clearly indicating the presence of the drug. It can also be seen that the shape and size of the neat aerogels does not change. In a previous study, supercritical impregnation was proven to be a completely safe method for the impregnation of drugs into alginate aerogels [41]. Only alginate aerogels were chosen as representative for the esomeprazole impregnation, although both pectin and alginate aerogels have the same shape, size, and appearance.

### 3.3. In Vitro Dissolution Tests

In vitro dissolution tests were performed for esomeprazole in its pure, crystalline form and the results were compared to various bioaerogel samples with impregnated esomeprazole. One aim of this study was to investigate the difference between pure esomeprazole and esomeprazole impregnated within various bioaerogel samples. However, the main objective was to investigate the influence of various carriers and two different impregnation methods on the release and dissolution profiles of esomeprazole.

Therefore, in vitro dissolution tests were performed in both SGF and SIF. The tested samples were first placed in SGF for 2 h and then transferred to SIF for an additional 22 h. The experiments were performed in a matter that imitates the oral pathway of the drug through the human body. Once the drug enters the stomach, it stays there for 1–2 h before moving to the intestine for an additional 7–22 h on average, depending on the intestinal emptying.

**Figure 6:**
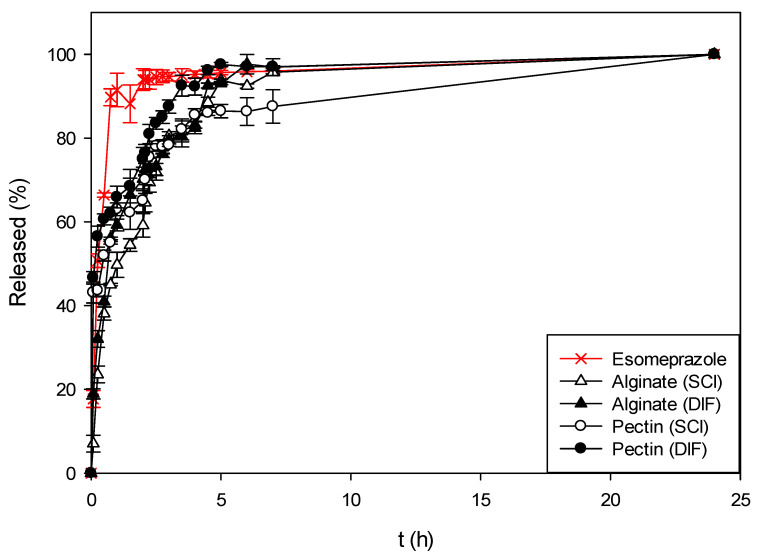
Dissolution profiles of pure esomeprazole and esomeprazole impregnated into neat alginate and pectin aerogels using both impregnation techniques.

**Figure 7:**
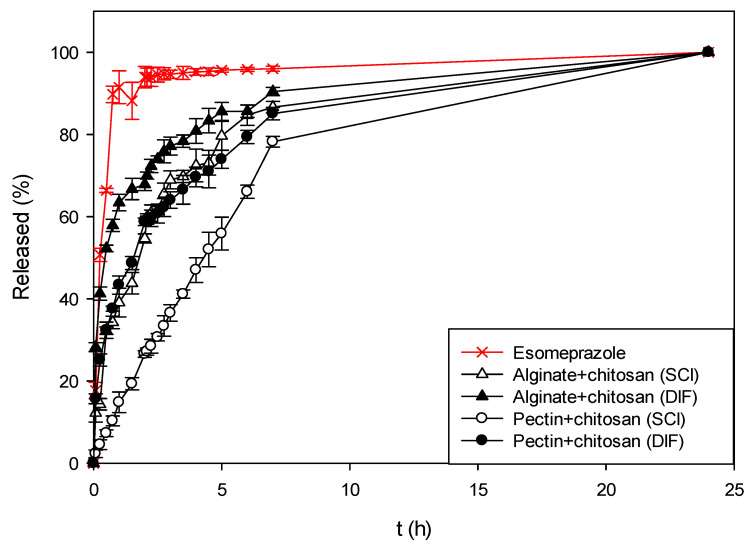
Dissolution profiles of pure esomeprazole and esomeprazole impregnated into alginate and pectin aerogels coated with chitosan using both impregnation techniques.

**Figure 8:**
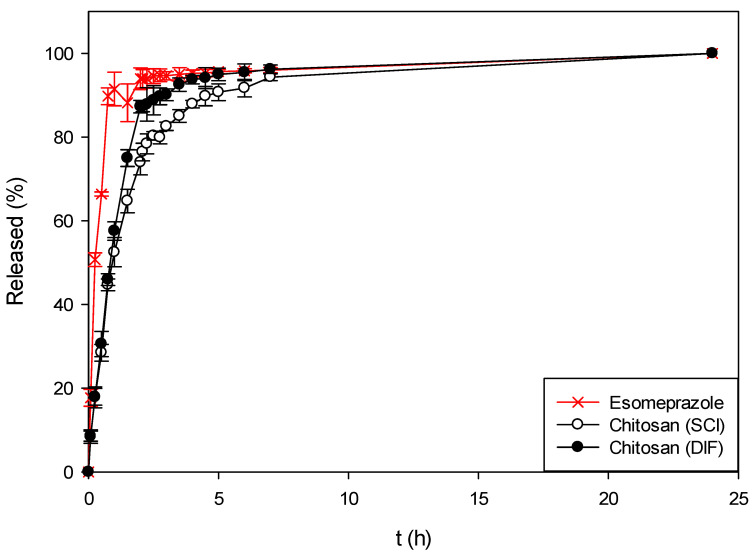
Dissolution profiles of pure esomeprazole and esomeprazole impregnated into chitosan aerogels using both impregnation techniques.

In this study, the dissolution tests were first performed for esomeprazole in its pure, crystalline form. The dissolution profile is presented in Figure 6, Figure 7 and Figure 8 with a red line, as a comparison to all the impregnated esomeprazole from various samples.

In the first part, the release of pure esomeprazole and esomeprazole impregnated into neat alginate and pectin aerogels was compared. As presented in Figure 6, approximately 94 ± 3% of pure esomeprazole was released within the first 2 h in SGF. The profile presented for esomeprazole is representative of a burst release. This means that as soon as it enters the stomach the drug dissolves very quickly in a contact with low pH, leading to almost complete dissolution within the first hour. When esomeprazole was impregnated into either alginate or pectin aerogels, a clear difference can be seen. This difference is especially visible in the SGF part, in low pH ranges. When impregnated into either alginate or pectin aerogels, the release of esomeprazole was visibly slowed down. In the case of alginate aerogels, 60–70% of esomeprazole was released in SGF within the first 2 h, depending on the impregnation method used. In the case of pectin aerogels, these percentages are slightly higher and are around 65–75%. However, in both cases the influence of the impregnation technique on the dissolution profiles is clearly seen. In this, a slower release was achieved with the samples when the supercritical impregnation technique was used as a loading method. Consequently, the percentage of released esomeprazole in SGF was lower and partially transferred to the SIF. The slowest release was observed for esomeprazole impregnated into pectin aerogels using the supercritical impregnation method. By impregnating esomeprazole into neat alginate and pectin aerogels, the release and consequently dissolution of esomeprazole was retained by approximately 20–30% in SGF.

Following the coated composite aerogels samples, the influence of the chitosan coating on the dissolution profiles was investigated. As presented in Figure 7, when esomeprazole was impregnated with alginate and pectin aerogels coated with chitosan, the release of esomeprazole was even more slowed down. This behavior was expected, since the main reason for coating the neat aerogels was to achieve a slower release of drugs in a more controllable matter.

In the case of alginate aerogels coated with chitosan, 55–70% of esomeprazole was released within the first 2 h in SGF. When using pectin aerogels coated with chitosan, this percentage was even lower, reaching 30–70%. Having a chitosan coating over the alginate or pectin core significantly lowers the release and slows down the dissolution of esomeprazole. The amount of esomeprazole released was also influenced by the impregnation technique used. The same phenomena were observed for both alginate and pectin aerogels coated with chitosan, as for the neat aerogels. When esomeprazole was impregnated using the supercritical impregnation technique, its release from the aerogels was slower compared to the release of esomeprazole impregnated via ethanol. This difference is even more profoundly observed for coated aerogels. Again, the slowest release was observed for pectin aerogels, in this case having a chitosan coating, where only 30% of the whole esomeprazole content was released in SGF. For this specific case, a controlled release of esomeprazole during the whole tested period of 24 h was achieved. Finally, when esomeprazole was impregnated into the coated aerogels, the release of esomeprazole compared to the pure form was retained by 30–60%, and by 30% compared to the esomeprazole impregnated within the neat alginate and pectin aerogels.

In the last step, chitosan was tested as the carrier for esomeprazole. As can be seen from Figure 8, by impregnating esomeprazole into chitosan aerogels using any of the mentioned techniques the release and dissolution of impregnated esomeprazole was slowed down to the pure form. Here, the same trend of slowing down the esomeprazole release was observed for chitosan as well as for alginate and pectin aerogels. When using chitosan, 75–85% of esomeprazole was released within the first 2 h in SGF. A faster release was achieved compared to alginate or pectin and alginate or pectin aerogels coated with chitosan. This behavior was expected since chitosan is a polysaccharide insoluble in water, but soluble in acidic medium. The chitosan partly dissolved in SGF leading to a faster release and dissolution of esomeprazole within the first 2 h. Furthermore, the same trend for esomeprazole impregnated via supercritical fluid is present, having a slower release rate compared to the esomeprazole impregnated via ethanol. Compared to the pure esomeprazole, using chitosan aerogels, the release was retained by 10–20%. 

## 4. Discussion

In this study, it was found that in all cases the highest loadings were achieved when the diffusion from ethanol was used as the impregnation technique. However, the loadings achieved in both cases were comparable. On the one hand, using diffusion from ethanol is a process that goes on simultaneously with the preparation and synthesis of bioaerogels, and thus a significant time is saved. In contrast, when supercritical impregnation is performed additional time is required to carry out the impregnation of drugs. However, significantly lower quantities (milligrams compared to grams) of the drug are used to achieve a similar loading as for diffusion from ethanol. This is especially important for expensive drugs, where waste time must be kept to a minimum.

The burst release of esomeprazole in the stomach is slowed down using any of the bioaerogels examined in this work. The main problem with a very fast dissolution of esomeprazole in the stomach is that it leads to lower bioavailability (up to 64%) [20]. By impregnating esomeprazole into bioaerogels, its release and therefore dissolution is influenced by the nature of the polysaccharide. For chitosan aerogels, the release of esomeprazole was slightly faster compared to that seen with the alginate or pectin aerogels. While alginate and pectin are insoluble in acidic medium, chitosan is soluble which led to the dissolution profiles shown in this work. The release of esomeprazole was the slowest in the case of coated aerogels. The release of esomeprazole was furthermore influenced by the impregnation technique used. In all cases, the supercritical impregnation led to a slower release and dissolution of esomeprazole. With both techniques, diffusion from ethanol and supercritical impregnation, the leading mechanism of impregnation is diffusion. In the first case, the diffusion occurs via ethanol and in the latter via supercritical CO_2_. One possible explanation for such a behavior could be that by impregnating esomeprazole via supercritical CO_2_, the impregnation goes deeper, capturing the lowest and the farthest pores present in the aerogel network. This leads to a slower release, and consequently delayed dissolution of the tested drug.

However, the slowest release is not always the most desirable release. This depends on the drug, the nature of the drug, and the target for its release. Since esomeprazole is a drug used to reduce gastric acid, it is desirable that most of it is released in the stomach, and in a controllable manner. By impregnating esomeprazole into any of the mentioned bioaerogels, a more controlled release was achieved. However, in the case of coated aerogels, and considering the nature and the target release of esomeprazole, they would not be appropriate carriers for this specific drug. On the other hand, chitosan aerogels showed more controlled release compared to pure esomeprazole, while not too slow. In this case a great amount of esomeprazole is released in the stomach in a controllable manner, which could significantly improve its bioavailability. A smaller part, 15–25%, is transferred to the intestine, which can thus be used to treat syndromes such as peptic ulcers that are also found in the upper part of the intestinal tract.

## 5. Conclusions

To conclude, the study presented here included the preparation and synthesis of various bioaerogels and blends thereof. Two different approaches were used for the impregnation of esomeprazole, diffusion from the ethanol solution and supercritical impregnation. Diffusion from the ethanol solution led to higher loadings, while the supercritical impregnation offered similar loadings with a significantly lower quantity of the drug used for the process.

In vitro dissolution tests revealed that by impregnating esomeprazole into any of the used bioaerogels, using either of the two approaches, improved the release and the dissolution profiles were achieved compared to the pure esomeprazole. Moreover, the controlled release of impregnated esomeprazole was achieved. The slower release was observed when supercritical impregnation was used for all the tested samples. The release of esomeprazole was also influenced by the polysaccharide used (alginate, pectin or chitosan) and the type of bioaerogel (neat or coated). For the tested drug, i.e., esomeprazole, chitosan aerogels were found to be the best choice. The release of esomeprazole was retained by up to 25% compared to that seen with pure esomeprazole and transferred to the intestine. Most importantly, the controlled release was achieved, significantly improving the chances of improved bioavailability.

## Figures and Tables

**Table 1:** The specific surface areas of pectin, alginate, chitosan, and their composites.

Sample	S_BET_, m^2^/g
Pectin	501 ± 6
Alginate	220 ± 10
Chitosan	431 ± 11
Pectin + chitosan	314 ± 8
Alginate + chitosan	248 ± 12

**Table 2:** Achieved loading for pectin, alginate, chitosan, and their composites.

Bioaerogel	Loading	
	SCI	DIF
Pectin	16.5 ± 1.0	19.5 ± 2.0
Alginate	10 ± 0.5	11.5 ± 0.5
Chitosan	15.5 ± 1.5	22 ± 1.5
Pectin + chitosan	2.5 ± 0.5	4 ± 0.5
Alginate + chitosan	9 ± 0.5	8.5 ± 0.5
